# The Effectiveness of Germany’s Compulsory Forensic Addiction Treatment: A Quasi-Experimental Study of Recidivism Using Matched Groups

**DOI:** 10.1177/0306624X251388483

**Published:** 2025-11-09

**Authors:** Norbert Schalast, Bastian Nau, Joscha Hausam, Johannes Fuss

**Affiliations:** 1 Institute of Forensic Psychiatry and Sex Research, Center for Translational Neuro- and Behavioral Sciences, University of Duisburg-Essen, Germany; 2 Institute of Forensic Psychiatry, Charité – Universitätsmedizin Berlin, Germany

**Keywords:** criminal offender, offender treatment, substance use disorder, involuntary treatment, imprisonment, matched controls, recidivism, forensic psychiatry

## Abstract

Substance use disorders are highly prevalent among offenders and are closely associated with increased rates of recidivism. This service evaluation assessed the effectiveness of compulsory forensic treatment for offenders with substance abuse in reducing recidivism. The study compared recidivism rates of 279 offenders who received mandated treatment under Section 64 of the German Criminal Code with those of a matched control group of 274 incarcerated individuals. An additional propensity score analysis confirmed the adequacy of the case-to-case matching. Over a mean follow-up of 6.5 years, the absolute risk reduction remained stable at around 16.5%, with recidivism rates at 7 years of 63.4% in the treatment group and 80.3% in the prisoner group. These findings attest to the effectiveness of compulsory addiction treatment in reducing recidivism compared to subjects who only serve a prison sentence, even though a substantial number (48%) of patients had been returned to prison but were still included in the treatment group at follow-up (intention-to-treat-analysis). The practical implications of these findings are discussed in light of recent legislative changes affecting the application of Section 64.

## Plain Language Summary

The effectiveness of Germany’s court-ordered addiction treatment for offenders: A study comparing reoffending rates in matched groups

Many people who commit crimes are addicted to alcohol or drugs, which increases their chances of committing new crimes after release. This study looked at whether a specific, court-ordered treatment program in Germany can help reduce reoffending. The program is known as Section 64 of the German Criminal Code, and is used to provide mandatory addiction treatment instead of a regular prison sentence. To find out how effective this treatment is, researchers compared two similar groups of offenders: 279 people who took part in the treatment and 274 who served regular prison sentences. The groups were matched closely to make sure they were similar in important ways. A statistical method called propensity score analysis was used to confirm that the matching was appropriate. After an average of 6.3 years, the study found that those who had received treatment were less likely to commit new crimes. About 63% of them reoffended, compared to 80% in the prison group, a stable difference of about 16.5 percentage points. This positive result appeared even though nearly half of the people in the treatment group were sent back to prison during the treatment. These individuals were still included in the analysis. The findings suggest that court-ordered addiction treatment in Germany can help reduce repeat offending. They are also relevant for current discussions about changes in the law affecting how this type of treatment is used.

## Introduction

### Substance Use Disorders and Offending

There is a relevant link between the use and abuse of intoxicants and delinquent behavior (Bennett et al., 2008). When committing offences, people are often intoxicated, and the prevalence of substance use disorders in prisons is high. Fazel et al. (2017) presented findings from a meta-analysis of 24 studies with a total of 18,388 recently incarcerated prisoners across 10 countries. The prevalence of alcohol use disorders among male prisoners ranged from 16% to 51% and of drug use disorders from 10% to 61%. Moreover, the meta-analysis indicated a rise in the prevalence of drug use disorders among prisoners throughout the preceding decades.

Excessive alcohol and drug use contributes to the risk of violent behavior (Duke et al., 2018; Tomlinson et al., 2016; Zhong et al., 2020), and financing illicit drug use is often associated with low-income acquisitive crime (Hayhurst et al., 2013). After adolescence, illicit drug use is particularly associated with serious violent crime (Fazel et al., 2018; White et al., 2015; Whiting et al., 2021).

Health care systems provide a range of treatment services for people with substance use disorders, but many drug users are reluctant to seek help and to accept treatment. However, given the established evidence that substance use is a significant predictor of reoffending in prison inmates (O’Driscoll et al., 2012; Young et al., 2011), public safety may require that the risk posed by drug-users be minimized through appropriate treatment. Therefore, many criminal codes provide for compulsory treatment of addicted offenders (Israelsson & Gerdner, 2012). In Germany, this is addressed in Section 64 of the Criminal Code.

### Findings Regarding the Benefits of Criminal Justice Interventions

In 2018, de Waard published a systematic overview of meta-evaluations / synthesis studies within the knowledge domains of Situational Crime Prevention, Policing, and Criminal Justice Interventions, including 219 meta-analyses focusing criminal justice interventions. Overall, there appeared to be a 12 % difference in reoffending rates between supported and comparison groups. Thus, on average, criminal justice interventions seemed to prevent one in eight cases of reoffending. De Waard highlights that treatment effects decline the more the intervention gets removed from the concrete behavior and the conditions in which that behavior occurs: *“If the social context is ignored, it will be more likely that the intended effects will not be achieved.” (p. 13).*

Werb et al. (2016) conducted a systematic review of studies evaluating the outcomes of compulsory treatment. Of the 430 studies initially identified, only nine met the authors’ strict inclusion criteria. While three studies reported no significant effects compared with control interventions, two studies found equivocal results (albeit without a control condition), and two studies found negative effects on recidivism. Only two studies found some positive effect of compulsory treatment on recidivism and drug use; one of these reported illegal drug use within 1 week of release from compulsory treatment (Strauss & Falkin, 2001). According to the other study (Hiller et al., 2006), those who dropped out of a program had a higher risk of reoffending than those who succeeded. Werb et al. (2016) thus concluded that there is very little evidence to support the concept of compulsory treatment in prison. The results of other studies also have been equivocal; compulsory treatment has been shown to have a positive impact on recidivism in some cases, and no impact in others, while in certain instances it has even been shown to have a negative effect (Farabee et al., 1998; Klag et al., 2005).

### The System of Offender Treatment According to Section 64 of the German Penal Code

When this study was conducted, the prerequisites for a Section 64 sentence were as follows: (i) a habit or tendency to use alcohol or other drugs excessively; (ii) an offence or offences related to this habit; (iii) a risk of further serious offences due to the habit; and (iv) reasonable prospects of successful treatment to prevent further offences. The explicit purpose of Section 64 is risk reduction. The legislation cannot be used to ameliorate a person’s problematic health condition if he or she is not “sufficiently dangerous” and/or if a treatment is unlikely to be successful. Courts regularly seek the opinion of a (psychiatric or psychological) expert to form their judgement on the above conditions.

While specific interventions may vary across institutions, the treatment generally comprises psychotherapy, vocational training, sporting activities, and assistance in planning for the future. It is noteworthy that the utilization of substitution therapy for the treatment of substance dependencies remains infrequent within these institutions. Treatment facilities under Section 64 have historically concentrated on abstinence-based approaches, with the consequence that in 2019, only approximately 6.3% of patients received substitution therapy (Soyka & Steinböck, 2022).

The treatment takes place in specialized facilities providing a certain degree of security. All the institutions treating these patients provide at least one high security ward or building for patients recently admitted or for crisis intervention. After a period of stable cooperation, patients usually change to medium- or low-security wards. The duration of treatment in a Section 64 facility is a priori limited. In principle, the maximum duration of treatment is 2 years, but an additional prison sentence (which is the regular case) increases the possible duration. In practice, the mean duration of treatment is around 2 years. This may include a prolonged period of outpatient treatment: many patients live in the community for months before being formally discharged by court order, with support and supervision from hospital staff.

Whenever a patient is formally discharged from treatment, the court will order a “supervision of conduct” (acc. to Section 68 of the Penal Code). This allows to issue a variety of instructions to a discharged patient (or prisoner), e.g. regarding substance use, avoiding specific persons and locations, accepting additional treatment or medication, making efforts to find and keep an occupation and others. Instructions may be enforced by penalty, if they are closely linked to the risk of reoffending. The supervision of conduct may be ordered for 2 to 5 years, in case of Section 64 patients frequently for 5 years. This also refers to patients who were returned to prison and are discharged from there.

However, treatment is often terminated because it is no longer seen as likely to lead to success. In such cases, patients are returned to prison. Such decisions must be made by a district court. The rate of early termination of treatment due to poor prospects of success has reached almost 50% in recent years. It tends to be higher when institutional capacity is under pressure. Moreover, there has been a considerable increase in the number of orders for treatment under Section 64 in recent years. For instance, between 2009 and 2019, the annual number of such orders increased by 52.4%, rising from 2,176 to 3,317 (Deutscher Bundestag, 2021). As a result, there has been an ongoing debate about the appropriateness and the rehabilitative benefits of the measure leading to a revision of Section 64 in October 2023.

### Findings Regarding the Benefit of Section 64 Treatments

Research on the effectiveness of forensic addiction treatment in Germany has yielded equivocal results. A number of reviews by Querengässer et al. (2015) and Querengässer and Bauer (2021a, 2021b) investigated factors predictive of treatment outcome under Section 64. The authors of the study identified that individual factors, including personality disorders and a criminal background, were significant predictors of treatment outcome. The combination of early onset of delinquency, problematic social and/or occupational background and specific personality components was found to indicate a high risk of premature dismissal from treatment and return to prison. Additionally, a recent systematic review by Tomlin et al. (2024) sought to provide a comprehensive overview of the evidence regarding mandatory substance use treatment for justice-involved persons in Germany. The authors of the study found that while some programs showed positive outcomes, others had negative impacts on criminal recidivism. At the program level, more effective programs were found to be those that ensured a continuum of care across the criminal justice system. However, the review also emphasized the necessity for additional research to clarify the impact of different treatments on social adjustment and to identify effective predictors of treatment outcome.

These reviews provide some evidence on the impact of different types of treatments and potential influencing factors for positive or negative outcomes. However, they also reveal a central research gap: it remains unclear whether treatment under Section 64 generally improves the social adjustment of patients compared to prisoners with a comparable background. Addressing this gap, the present comprehensive service evaluation was designed to examine whether Section 64 orders achieve their intended purpose of reducing recidivism. The Ministry of Health of North Rhine-Westphalia (NRW) agreed to fund this evaluation study.

### The Present Service Evaluation

This study concludes a series of investigations into the effects of treatment on individuals subjected to Section 64 orders. The first preliminary request for follow-up data was submitted to the relevant authorities in 2016. Based on these data, initial analyses demonstrated positive treatment effects for men (Schalast, 2021) and women (Frey, 2019) over a 4-year follow-up period. In a subsequent publication (Schalast et al., 2021), we addressed methodological criticisms raised in the literature (e.g. Müller, 2021), particularly concerns regarding the comparability of the study groups. The present analyses build on this earlier work and extend it in two important respects: first, by examining an extended follow-up period of on average 6.5 years, based on a second request for follow-up data in 2019; and second, by applying statistical matching procedures to improve and ensure comparability between the groups. The length of the follow-up period in this study is of particular significance, because the follow-up period in the previous evaluation ended before the supervision of conduct (acc. to Section 68 of the Criminal Code) ended.

The aim of the present study was to compare patients under Section 64 orders and a carefully matched sample of prisoners in terms of reoffending after release. We hypothesize that patients under Section 64 are significantly less likely to reoffend after discharge than prisoners. Furthermore, we hypothesize that patients who successfully complete the program (positive outcome) are significantly less likely to reoffend than those who drop out and are returned to prison (negative outcome).

## Method

### Procedure

Data collection was conducted in 16 forensic psychiatric facilities housing patients under Section 64 of the German Criminal Code (StGB), as well as in 15 correctional institutions. The approach varied slightly across settings. In the forensic psychiatric facilities, data was collected by clinical staff using a structured assessment scheme that provided basic information about patients’ characteristics, such as age and index offense. They also reported on the duration and outcome of treatment (i.e., discharge to the community or return to prison). All patients admitted to the hospitals between October 2009 and December 2010 were included in the evaluation. Patients were informed in writing about the project and could contact hospital staff and the NRW data protection officer in case of questions or concerns. Members of the project team did not meet patients, had no access to records, and had no influence whatsoever on treatment decisions.

The prison sample was recruited between 2011 and 2012, with the help of the social services within the prisons. Case-to-case matching was based on five risk-relevant characteristics, according to which the “prison twin” was identified: age at admission (< 25; 25–35; 36–50; > 50), predominant substance use (mainly alcohol; multiple substances including illegal drugs), number of previous convictions (< 5; 5–10; > 10), prior imprisonment due to an earlier sentence (yes; no), and offense category (general violence; sex offense; property offense; drug offense; other). The rather broad categorization of “predominant substance use” reflects routine clinical practice in prison and forensic settings. The characteristics employed in this study, i.e., age (Katsiyannis et al., 2018), type of substance use (Yukhnenko et al., 2020), previous convictions (Bushway et al., 2011; Gendreau et al., 1996), previous prison sentences (Bales & Piquero, 2012) and offence category (Craig et al., 2006) have been identified as significant factors for the risk of reoffending.

Within this service evaluation, data collection was carried out exclusively using pseudonyms. In order to protect the identity and privacy of the individuals involved, re-identification for the purpose of obtaining criminal records from the Federal Central Register was conducted by the State Commissioner for Forensic Psychiatry, an authority within the Ministry of Health of NRW. The criminal records were requested in April 2019 from the Federal Office of Justice. Noteworthy, every misspelling or incorrect digit (e.g., on the date of birth) in the data transmitted to the authority resulted in an empty “negative report.” Due to the procedure, such errors could not be corrected retrospectively. Furthermore, the Federal Office of Justice does not provide information on deceased individuals; as a result, these cases had to be excluded from the analysis.

### Material

Criminal records were obtained after an average follow-up period of 6.5 years (*M* = 2.306 days; *SD* = 415). The follow-up period (time at risk) was defined as the period from the formal discharge of a patient or prisoner into the community to the date of the information provided by the Federal Office of Justice. There was no significant difference between the time at risk for patients (*M* = 2.320, *SD* = 457) and prisoners (*M* = 2.291, *SD* = 367), *t*(530.48) = 0.799, *p* = .425, *d* = .07(Table 1).

**Table 1.** Statistics of the Follow-Up Times in Both Samples (Days).

Sample	*N*	Mean	*SD*	Median	Min.	Max.
Art. 64 treatment	279	2320	457	2357	599	3280
Prison	274	2291	367	2361	1067	3020
Total	553	2306	414	2360	599	3280

For clarity, the values have been rounded to whole numbers.

Based on the criminal records, recidivism after release was assessed by recording every new conviction. In addition, the individuals’ criminal histories prior to the index offense were systematically assessed. This step aimed to examine potential differences in criminal history – a well-established risk factor for recidivism (Bushway et al., 2011; Gendreau et al., 1996) – between the groups. The following indicators of prior criminal history were recorded: the number of sentences under juvenile and adult law, the total number of recorded offenses, the number of violent offenses, the number of offense categories, the number of prison sentences, and the total duration of prison sentences.

### Sample

A total of 315 male patients and 315 male prisoners were initially recruited. A separate evaluation focusing on women under Section 64 treatment was conducted by Frey in 2019. Due to missing data, 36 patients and 41 prisoners had to be excluded, resulting in a final sample of 279 patients and 274 prisoners. One reason for missing data was the death of patients and prisoners. During the follow-up period, 19 individuals from the original prisoner sample (6.0 %) and six individuals from the original patient sample (1.9 %) passed away and were excluded from the evaluation. The fatality rates in both samples differed significantly (*χ*^2^(1) = 6.701, *p* = .010).

The mean age of the final sample in the year 2012 was 35.81 years (*SD* = 8.34). There was no significant age difference between the two groups, *t*(544) = −1.441, *p* = .150, *d* = −.12. The mean age of the patients was 35.29 years (*SD* = 8.48), while the mean age of the prisoners was 36.32 years (*SD* = 8.17).

Table 2 presents the distribution of index offenses across both groups. No significant group differences were found (*χ*^2^(7) = 7.96, *p* = .336).

**Table 2.** Distribution of Index Offences.

Offence category	Patients		Prisoners		Total	
	*N*	%	*N*	%	*N*	%
(Attempted) Homicide	10	3.7	7	2.6	17	3.1
Assault and battery	57	21.0	59	21.9	116	21.5
Sexual offence	6	2.2	9	3.3	15	2.8
Theft, robbery, fraud	95	35.1	98	36.4	193	35.7
Hostage-taking, kidnapping	1	0.4	1	0.4	2	0.4
Arson	7	2.6	2	0.7	9	1.7
Drug-related crime	79	29.2	66	24.5	145	26.9
Other offences	16	5.9	27	10.0	43	8.0

The mean duration of treatment for patients was 111.87 weeks (*SD* = 57.75). A considerable number of patients live in the community for months before being formally discharged, with ongoing support and supervision from hospital staff. Therefore, on average 17.42 weeks (*SD* = 22.05) of this treatment period was carried out as outpatient treatment.

Nearly half of the patients (48.3%; *n* = 135) discontinued treatment prematurely and were transferred back to prison. For the main analyses, they were retained in the patient group following an intention-to-treat approach.

### Group Equivalence

In observational studies, ensuring group equivalence is crucial in order to minimize bias and improve the ability to derive meaningful findings and conclusions. This means that observed differences in relapse rates can be attributed to the intervention rather than to pre-existing group differences. There were no significant differences in criminal history between the groups (see Table 3). Thus, the case-to-case matching appears to have been effective in ensuring the equivalence of the two groups.

**Table 3.** Distribution of Some Criminological Characteristics in the Samples (Crime Register Data).

Criminological characteristics	*M*		*SD*		Test-statistic		
	Patients	Prisoners	Patients	Prisoners	*t (df)*	*p*	*d* ^c^
Age in the year 2012	35.29	36.32	8.48	8.17	−1.441(544)	.150	−.12
Times sentenced (juvenile law)	3.21	3.23	3.23	3.05	−.074(534)	.941	−.01
Times sentenced (general law)	6.42	6.99	5.34	5.08	−1.265(534)	.206	−.11
Total no. of offences recorded	31.38	29.30	32.21	30.21	.773(533)	.220	.07
No. of violent offences	2.52	2.27	3.10	2.84	.950(525)	.343	.08
No. of offence categories^a^	4.12	4.13	1.73	1.65	−.051(524)	.959	.00
No. of prison sentences	4.43	4.95	4.10	3.88	−1.506(533)	.133	−.13
Σ Prison sentences (months)^b^	51.75	52.29	52.33	42.25	−.131(499.651)	.896	−.01

a Criminal diversity (number of offence categories – out of 14 categories).

b Sum of ordered prison sentences (not necessarily served completely).

c Cohen’s d.

Although the case-to-case matching already supported group comparability, additional statistical matching and weighting procedures were applied to further validate this equivalence (Greifer & Stuart, 2021). Put simply, these procedures allow for the assessment of covariate balance and enable subsequent statistical analyses to be conducted using weighted or matched samples. We selected the variables used in the case-to-case matching, along with the indicators of criminal history as covariates. Weighting procedures generally indicated better covariate balance than matching procedures. For illustration purposes here, we applied a simple propensity score weighting approach. Covariate balance was assessed using the most commonly applied metric: absolute standardized mean differences (ASMD). Guidelines suggest that values below 0.1 indicate good balance, values up to 0.25 are acceptable, and values greater than 0.25 suggest insufficient similarity for valid group comparisons (Stuart et al., 2013). The weighting procedures improved covariate balance, and all ASMD values were below 0.1, with the exception of “total no. of offences recorded” (ASMD = 0.119).

Finally, we examined the extent to which the results of the unweighted and weighted analyses differed. As the findings were nearly identical, we concluded that it was reasonable to retain the unweighted analyses presented in the manuscript, as they offer a more straightforward and accessible interpretation for readers.

### Statistical Analyses and Power Analysis

Statistical analyses were performed using IBM SPSS Statistics 29.0. Kaplan and Meier (1958) survival analysis (time-to-event analysis) was used to compare recidivism of patients and prisoners at follow-up. Survival analysis offers the advantage of accounting for varying follow-up times and censoring, making it particularly suitable for examining time until reoffending in longitudinal data. Mantel Cox regression was used to examine the association between several variables and the frequency of re-offending during the time at risk. Assumptions for Cox regression were assessed using Schoenfeld residuals and survival plots; no violations of proportionality were detected. In addition to the intention-to-treat analyses, treatment dropouts were examined in more detail. For this purpose, three groups were formed and analyzed separately.

We conducted a post hoc power analysis (Schoenfeld method) using our final sample of 553 participants (Group 1: *n* = 279; Group 2: *n* = 274) and 397 observed events. This analysis indicated that the study had >99% power (two-sided *α* = .05) to detect the observed between-group effect.

## Results

**Figure 1.**
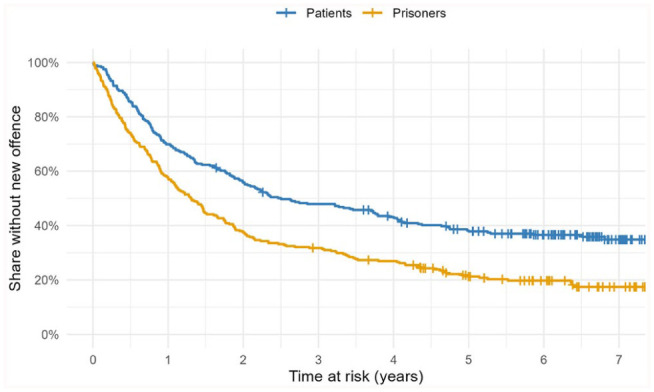
Kaplan-Meier survival analysis of Federal Crime Register data for both samples. Criterion: any new entry indicating a new sentenced offence. Statistic: percentage of individuals without new entry during the time at risk (Kaplan-Meier estimates). Groups differ significantly (log rank chi² = 24.54, *df* = 1, *p* < .001); small vertical lines: censored cases (follow up ended before a new entry occurred).

Figure 1 shows the results of a survival analysis of the two samples. Patients had significantly fewer new entries in the Criminal Record compared to the prisoners (*Log Rank χ*^2^(1) = 24.54, *p* < .001). The graph lines in Figure 1 describe the proportion (percentage) of individuals with no new entries in the criminal record over time. The small vertical lines in the graphs mark the end of the follow-up of individuals without a new entry (“censored cases”). Table 4 shows the annual rates of cases with no new entries in the criminal record. After 3 years of being at risk, the absolute risk reduction remains stable at around 16.5% for subsequent years.

**Table 4.** Percentage of Subjects without New Entry in the Federal Crime Register (Kaplan-Meyer-Estimates) After 1 to 7 Years “At Risk.”

	No new entries to the criminal record		Risk reduction	
Time at risk	Patients	Prisoners	Absolute RR (%)	Relative RR (%)
1 year	69.9% (195)	57.7% (158)	12.2	28.8
2 years	55.9% (156)	37.6% (103)	18.3	29.3
3 years	48.0% (134)	31.8% (87)	16.2	23.7
4 years	43.4% (121)	27.0% (74)	16.4	22.5
5 years	38.4% (107)	21.9% (60)	16.5	21.1
6 years	37.3% (104)	20.8% (57)	16.5	20.8
7 years	36.6% (102)	19.7% (54)	16.9	21.0

Absolute risk-reduction (Absolute RR) = difference between patients and prisoners; Relative risk-reduction (Relative RR) = difference divided by the share of cases with new entry in the prison-sample.

### The Impact of Positive and Negative Outcomes

**Figure 2.**
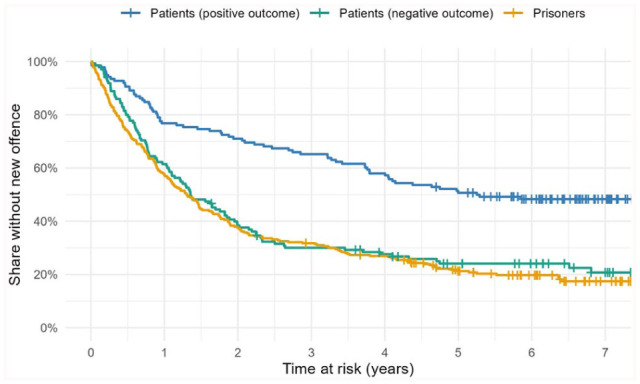
Kaplan-Meier survival analysis of Federal Crime Register data with the sample of patients split into subgroups “positive (finished treatment) vs. negative (returned to prison) outcome.” Criterion: any new entry indicating a new offence. Statistic: share of individuals without new entry during the time at risk (Kaplan-Meier estimates). Log rank chi² = 44.34, df = 2, *p* < .001); small vertical lines: censored cases (follow up ended before a new entry occurred).

After this intention-to-treat analysis, we divided the full sample into three subgroups for further statistical analysis: prisoners (*n* = 274), patients with positive (*n* = 138) and negative (*n* = 135) treatment outcomes (Figure 2). “Negative” means that patients were returned to prison and discharged after serving their sentence. All patients discharged directly into the community were categorized as “positive outcome.” Most of these patients received support in organizing their future life in the community, which included a period of several months living in their future environment before formal discharge. The “time at risk” begins with this formal discharge and not with the supervised leave from the institution.

First, we compared the two groups of patients, those with positive and negative outcomes, with respect to their previous criminal backgrounds. The only significant difference between the groups was the number of previous convictions under juvenile law (*t*(254) = −2.049, *p* = .041, *d* = −.26). There were no significant group differences in age (*t*(270) = .477, *p* = .634, *d* = .06), the number of sentences under adult law (*t*(254) = −.850, *p* = .396, *d* = −.01), the total number of recorded offenses (*t*(253) = −.347, *p* = .729, *d* = .04), the number of violent offenses (*t*(252) = −1.732, *p* = .084, *d* = −.22), the number of offense categories (*t*(251) = −1.887, *p* = .06, *d* = −.24), the number of prison sentences (*t*(253) = −1.624, *p* = .106, *d* = −.20), or the total duration of previous prison sentences (*t*(253) = −1.027, *p* = .306, *d* = −.13).

Subsequently, we examined the recidivism rates for new convictions among the three groups. After 7 years, patients with a positive outcome had no new entries in their criminal record in 48.6% of cases, patients with a negative outcome had no new entries in only about 23.7% of cases, and prisoners had no new entries in only about 19.7% of cases. Cox regression showed a significant overall effect (*Log Rank χ*^2^(2) = 44.34, *p* < .001). Pairwise Mantel Cox regression showed no significant difference between prisoners and patients with a negative outcome (*Log Rank χ*^2^ = .656, *p* = .418), patients with a positive outcome showed significant differences compared to prisoners (*Log Rank* χ^2^ = 43.314, *p* < .001) and patients with a negative outcome (*Log Rank χ*^2^ = 27.278, *p* < .001).

## Discussion

This study compared a large sample of forensic psychiatric patients (committed to compulsory addiction treatment under Section 64) with a matched sample of prisoners identified as heavy substance users, tracking their legal behavior over 6.5 years on average following their release. As intended by the matching process, the samples did not differ significantly in terms of age and criminological variables. It was further confirmed by an additional propensity score procedure that the matching process had been successfully conducted. The initial risk of reoffending could be considered similar and high in both groups.

Querengässer at al. (2018) demonstrated that patients who successfully complete compulsory forensic addiction treatment under Section 64 have lower recidivism rates than those who discontinue the treatment and are returned to prison. However, that evaluation did not test whether the treatment intervention in general has a significant impact on the risk of reoffending. In addition, the follow-up periods of studies researching a treatment effect of forensic addiction treatment on recidivism are frequently significantly shorter (de Andrade et al., 2018; Koehler et al., 2014; Werb et al., 2016). Our study monitored the legal behavior of patients and prisoners over a period of about 6.5 years on average after release into the community. As shown in Figure 1, Kaplan-Meier survival analysis demonstrates a highly significant difference in the trajectories of new entries to the criminal record, with a remarkably stable absolute risk reduction (fewer entries) of about 16.5 % in the patient sample after 3 to 7 years. This strong reduction in risk was observed even though patients with a negative treatment outcome – those who were returned to prison – remained in the patient sample in the first analysis. The second analysis between patients with a positive outcome and prisoners showed an even higher absolute risk reduction of 28.9%, while patients with a negative outcome had the same risk as prisoners. Although the negative outcome group tended to have a slightly more extensive criminal history compared to the positive outcome group, the only significant difference between the groups was the number of previous convictions under juvenile law. This finding highlights the constraints and challenges associated with the use of criminological characteristics in predicting the course of treatment under Section 64.

The focus of this service evaluation was on recidivism, as risk reduction is the purpose of Section 64 of the Criminal Code. To put it bluntly, if all patients continued to use drugs but stopped offending, the measure would fulfill its core purpose perfectly; if all patients stopped using drugs but continued to offend, Section 64 treatment would have failed according to the legal framework and would have to be abolished.

### Possible Explanations for the Effect Size of Forensic Addiction Treatments Under Section 64

There are multiple possible explanations for the effect size of compulsory addiction treatments according to Section 64: (i) Treatment does not take place in prison but in a special forensic treatment center. These centers are smaller than prisons and have better staff coverage, including nurses, psychologists, social workers and physicians. (ii) Section 64 treatments are lengthy: In their systematic review of studies evaluating the outcomes of compulsory treatment, Werb et al. (2016) found little evidence to support the concept of compulsory treatment in prison. However, in that review 6-month programs were the maximum; Section 64 patients are rarely discharged to the community before 18 months of treatment. (iii) The institutions provide a high level of security, but patients are regularly allowed to leave them unsupervised after a period of cooperation. (iv) The programs cover a wide range of activities such as work, sport, or psychotherapy. As treatment progresses, patients are given support in organizing their future lives, including subsistence, housing and aftercare. The treatment is thus strength-oriented and seems compatible with the *Good Lives Model* (Barnao et al., 2016; Ward & Brown, 2004) in many aspects. While earlier studies yielded inconclusive results (Farabee et al., 1998; Klag et al., 2005; Werb et al., 2016), the present study demonstrates that a time-intensive, appreciative and strength- and resource-oriented compulsory treatment can significantly and sustainably reduce criminal recidivism.

A substantial body of research has already demonstrated the efficacy of aftercare and reentry programs in reducing recidivism (Cannonier et al., 2021; James et al., 2013; McCollister et al., 2003; Olson & Lurigio, 2014). The discharge of a patient after a rather stable time in treatment always goes along with support in finding a job and an adequate place to live. Usually, patients leave the institution and begin to live and work outside the institution before their formal discharge. The treatment in the last months preceding the formal discharge can be characterized as a “coaching” in handling everyday problems and challenges. Regarding de Waard’s (2018) warning not to ignore the social context in the rehabilitating process one can state that the future social context of a patient is not ignored but a major focus of therapy and support in treatment under Section 64.

Additionally, Gwynne et al. (2020) asked 178 formerly incarcerated people to rate their quality of life after release. External circumstances (finances, support, and housing) proved more predictive of reoffending in the first year after release than subjective wellbeing (mental and physical health). Section 64 treatments have a strong focus on these external factors. It is therefore hardly surprising that in our service evaluation, patients with regular and supported release into the community have a much lower reoffending rate than prisoners and patients returned to prison.

### Drop-Outs and Treatment Termination

It is a common finding that “treatment drop-outs” do worse at follow-up and have a higher risk of reoffending (Kaskela & Pitkänen, 2021; Lockwood & Harris, 2015; Querengässer et al., 2018). It has been demonstrated that patients who discontinue therapy in various institutional treatment programs are frequently those with a high risk of recidivism, a particularly high need for therapy, and a high level of specific requirements (Brunner et al., 2019; Miller et al., 2004; Olver et al., 2011). Thus, the effect size of the German model of compulsory forensic addiction treatment would most likely be even larger if more patients received adequate treatment instead of being returned to prison. In their meta-analysis on offender addiction treatment, Koehler et al. (2014) state that “*it is clear that practitioners should invest as much effort as possible in maximizing the number of treatment completers.”* A low return rate to prison should be recognized as a criterion of good practice.

Looking at predictors of treatment outcomes, Querengässer et al. (2015, 2018) concluded that the specific institution receiving the patient is the best predictor. Treatment centers vary in their rates of treatment discontinuation and return to prison over time. An analysis of data from more than 30 forensic hospitals over 5 years showed that the “institution” factor accounted for 20% of the variance in outcomes (Schalast et al., 2021).

### Current State of Section 64

Efforts were made to tighten the legal presuppositions of a Section 64-sentence in Germany. Such a sentence used to be favorable for defendants as it allowed an earlier release from the institution in case of a positive course of treatment. The discharge was possible when half of the sentence was served by time in prison and treatment. If sentenced to imprisonment only, an offender can be released after serving two thirds of the sentence. This privilege made a Section 64-sentence attractive for defendants expecting a sentence of more than 2 or 3 years. This in turn contributed to a continuous increase in the number of patients, which put therapists and administrations under considerable pressure. A legislative reform abolishing such privileges became effective in October 2023. This reform also toughened the presuppositions of a respective sentence. Before the reform, “sufficiently concrete prospects” of a successful treatment could justify a Section 64 sentence. By now, it needs “factual indicators” promising a positive treatment outcome. As a result, individuals who are regarded as “difficult” or “unmotivated” may be increasingly referred to prison and not to Section 64 treatment. From the point of view of our findings, there is a need for treatment offers comparable to the established practice in Section 64 institutions. Schwarz and Stübner (2023) inspected documents on 70 former patients. Only one third of the sample seemed to fulfill the presuppositions for a treatment order according to the new regulation. The debate regarding an adequate way to deal with the co-occurrence of addiction and offending is still open. Our findings comparing reoffending rates of prisoners and of patients treated according to Section 64 (previous regulation) should be considered in this debate.

### Limitations

Within the framework of this service evaluation, detailed information about patients’ and prisoners’ past and follow-up substance use, German language proficiency, the severity of substance use disorders, and other sociodemographic variables was unavailable. Due to the absence of access to the subjects by the researchers, the evaluation was constrained to pseudonymized information provided by the prisons, forensic hospitals and the Federal Office of Justice. Future research might attempt to address this limitation by collecting information on those additional variables before and after release, which may also provide further insight into the causes of reoffending.

The subjects in our service evaluation were not assigned at random to interventions. Factually, random assignments to interventions such as prison and forensic treatment are for obvious reasons inconceivable. Nevertheless, we sought to ensure that the two samples were equivalent in terms of their initial risk of reoffending. The approach of using carefully matched groups seems to be the most viable and realistic way of comparing the effectiveness of Section 64 treatment with imprisonment. It should be noted, however, that this equivalence applies only to observed variables. One cannot exclude that unmeasured differences between the groups had some impact on recidivism. Additionally, recidivism was analyzed only as a global outcome. Distinctions between offense types (e.g., violent vs. non-violent) and offense severity (e.g., crime harm) would provide additional insights and should be addressed in future research.

## Conclusion

The findings of this study demonstrate that compulsory forensic addiction treatment under Section 64 in Germany significantly reduces the absolute risk of reoffending in comparison to a prison sentence. Survival analysis yielded a stable effect of the treatment over and above a mean observation period of 6.5 years. The substantial difference of reoffending in the follow-up period should thereby encourage to preserve – not to cut back – respective treatment options. Particular attention should be paid to increasing the number of treatment completers, which will most likely further increase the impact of the intervention.
